# An Increase in Reactive Oxygen Species by Deregulation of ARNT Enhances Chemotherapeutic Drug-Induced Cancer Cell Death

**DOI:** 10.1371/journal.pone.0099242

**Published:** 2014-06-12

**Authors:** Jiunn-Min Shieh, Chih-Jie Shen, Wei-Chiao Chang, Hung-Chi Cheng, Ya-Yi Chan, Wan-Chen Huang, Wen-Chang Chang, Ben-Kuen Chen

**Affiliations:** 1 Department of Internal Medicine, Chi Mei Medical Center, Tainan, Taiwan, ROC; 2 The Center of General Education, Chia Nan University of Pharmacy and Science, Tainan, Taiwan, ROC; 3 Institute of Bioinformatics and Biosignal Transduction, College of Bioscience and Biotechnology, National Cheng Kung University, Tainan, Taiwan, ROC; 4 Department of Clinical Pharmacology, and Master Program for Clinical Pharmacogenomics and Pharmacoproteomics, School of Pharmacology, Taipei Medical University, Taipei, Taiwan; Department of Pharmacy, Taipei Medical University-Wanfang Hospital, Taipei, Taiwan, ROC; 5 Institute of Biochemistry, National Cheng Kung University, Tainan, Taiwan, ROC; 6 Department of Pharmacology, College of Medicine, National Cheng Kung University, Tainan, Taiwan, ROC; 7 Graduate Institute of Medical Sciences, College of Medicine, Taipei Medical University, Taipei, Taiwan, ROC; Taipei Medicine University, Taiwan

## Abstract

**Background:**

Unique characteristics of tumor microenvironments can be used as targets of cancer therapy. The aryl hydrocarbon receptor nuclear translocator (ARNT) is an important mediator of tumor progression. However, the functional role of ARNT in chemotherapeutic drug-treated cancer remains unclear.

**Methodology/Principal Findings:**

Here, we found that knockdown of ARNT in cancer cells reduced the proliferation rate and the transformation ability of those cells. Moreover, cisplatin-induced cell apoptosis was enhanced in ARNT-deficient cells. Expression of ARNT also decreased in the presence of cisplatin through proteasomal degradation pathway. However, ARNT level was maintained in cisplatin-treated drug-resistant cells, which prevented cell from apoptosis. Interestingly, reactive oxygen species (ROS) dramatically increased when ARNT was knocked down in cancer cells, enhancing cisplatin-induced apoptosis. ROS promoted cell death was inhibited in cells treated with the ROS scavenger, N-acetyl-cysteine (NAC).

**Conclusions/Significance:**

These results suggested that the anticancer activity of cisplatin is attributable to its induction of the production of ROS by ARNT degradation. Targeting ARNT could be a potential strategy to eliminate drug resistance in cancer cells.

## Introduction

The aryl hydrocarbon receptor nuclear translocator (ARNT), also known as hypoxia-inducible factor (HIF)-1β, is a transcription factor that belongs to the basic helix-loop-helix Per-ARNT-Sim (bHLH-PAS) family, such as endothelial PAS domain protein 1 (EPAS1), HIF-1α, and aryl hydrocarbon receptor (AhR) [1]–[3]. The ARNT forms a heterodimer with HIF-1α in response to varying oxygen levels of microenvironments, and further promotes cell survival and angiogenesis [4]–[6]. In addition, disruption of ARNT in mouse embryonic stem cells causes hypoglycemia, an angiogenesis deficiency and a failure to respond to hypoxia [7]. Moreover, ARNT is a mediator in normoxic conditions when cells face harmful factors in the microenvironment, such as 2,3,7,8-tetra-chlorodibenzo[b,e][1], [4]-dioxin (TCDD) or anti-cancer drugs [8], [9]. The ARNT dimerizes with the aryl hydrocarbon receptor (AhR) and regulates Sp1 transcription activity, following upregulation of the promoter of cytochrome P450 subfamily polypeptide 1 (CYP1A1) to resist xenobiotic stresses, e.g., TCDD [3]. When regulating the ARNT in cells, it can be stabilized through interacting with the BRCA1 protein during TCDD stress [10]. On the other hand, active caspase-3 cleaves the ARNT during apoptosis to reduce cell survival signals [11]. Loss of HIF-1α and ARNT also leads to an increased response to radiotherapy, a reduction in tumor growth, and decrease in angiogenesis in tumors transplanted into immune-deficient mice [12]. In our previous studies, we found that ARNT interacted with c-Jun to form c-Jun/ARNT and c-Jun/ARNT/Sp1 complexes which promote expressions of cyclooxygenase (COX)-2, 12(S)-lipoxygenase, and p21*^WAF1/CIP1^*, in epidermal growth factor (EGF)-treated cervical cancer cells in a normoxic condition [1], [13]. Those studies indicated that ARNT interacts with HIF-1α in response to hypoxic conditions and also binds with specific transcription factors which have the ability to trigger the signaling of tumorigenesis in a normoxic condition.

Cisplatin is a major chemotherapeutic drug used with many kinds of cancers, especially testicular, ovarian, esophageal, cervical, gastric, prostate, and non-small-cell lung cancers [14]. After influx into a cell, cisplatin is hydrolyzed and becomes its active form. Crosslink of DNA strands occurs by active cisplatin binding to DNA on position 7 of guanine which causes cancer cell death [15], [16]. In addition to causing apoptosis by inducing cytochrome c release in response to DNA stress, cisplatin also induces cell apoptosis caused by reactive oxygen species (ROS) through a p53-mediated p38α mitogen-activated protein kinase (MAPK) pathway in HCT1116 colon cancer cells [17]. ROS are constantly generated and eliminated during normal physiological and biological functioning [18]. During oxidant stress, such as hypoxia or xenobiotic stimulation, ROS can be eliminated by scavenging proteins, such as superoxide dismutases (SODs) and glutathione peroxidase. Cancer cells exhibit greater tolerance of ROS than do normal cells. A low level of ROS facilitates cancer cells driving cell growth-associated genes, but a higher production of ROS also causes cells to undergo apoptosis [19]. However, cancer cells adapt to damage from cisplatin by upregulating efflux transporters, such as the ATP-binding cassette (ABC) transporter. Multidrug resistance 1 (MDR1) is a member of drug efflux ABC transporters that pump anti-cancer drugs outside of the membrane [20]. Moreover, overexpression of MDR1 allows human KB carcinoma cells to effectively resist colchicine, vinblastine, and doxorubicin [21]. Anti-cancer drugs cause cancer cells to acquire resistance through overexpression of drug-resistant genes. Therefore, understanding the molecular mechanism involved in regulating the acquisition of resistance in cancer cells would be beneficial to effective therapy.

In our previous study, we found that ARNT interacted with Sp1 to regulate MDR1 expression and protected cells from cisplatin-induced apoptosis [9]. The ARNT-regulated efflux of drugs was also observed in MDR1-upregulated cancer cells. These results reveal that ARNT is one of regulators to maintain cancer cells survival under cisplatin treatment. To further pursue the potential role of ARNT in maintaining cell survival, the effect of ARNT level on chemotherapeutic efficiency was studied in various cancer types. In this study, we found that the deregulation of ARNT not only reduced cell viability, but promoted cisplatin-induced cell death by enhancing the production of ROS. These results indicated that ARNT could simultaneously regulate MDR1 expression and reduce the ROS level to protect cancer cells from drug-induced apoptosis.

## Results

### The ARNT regulates cancer cell proliferation

**Figure 1:**
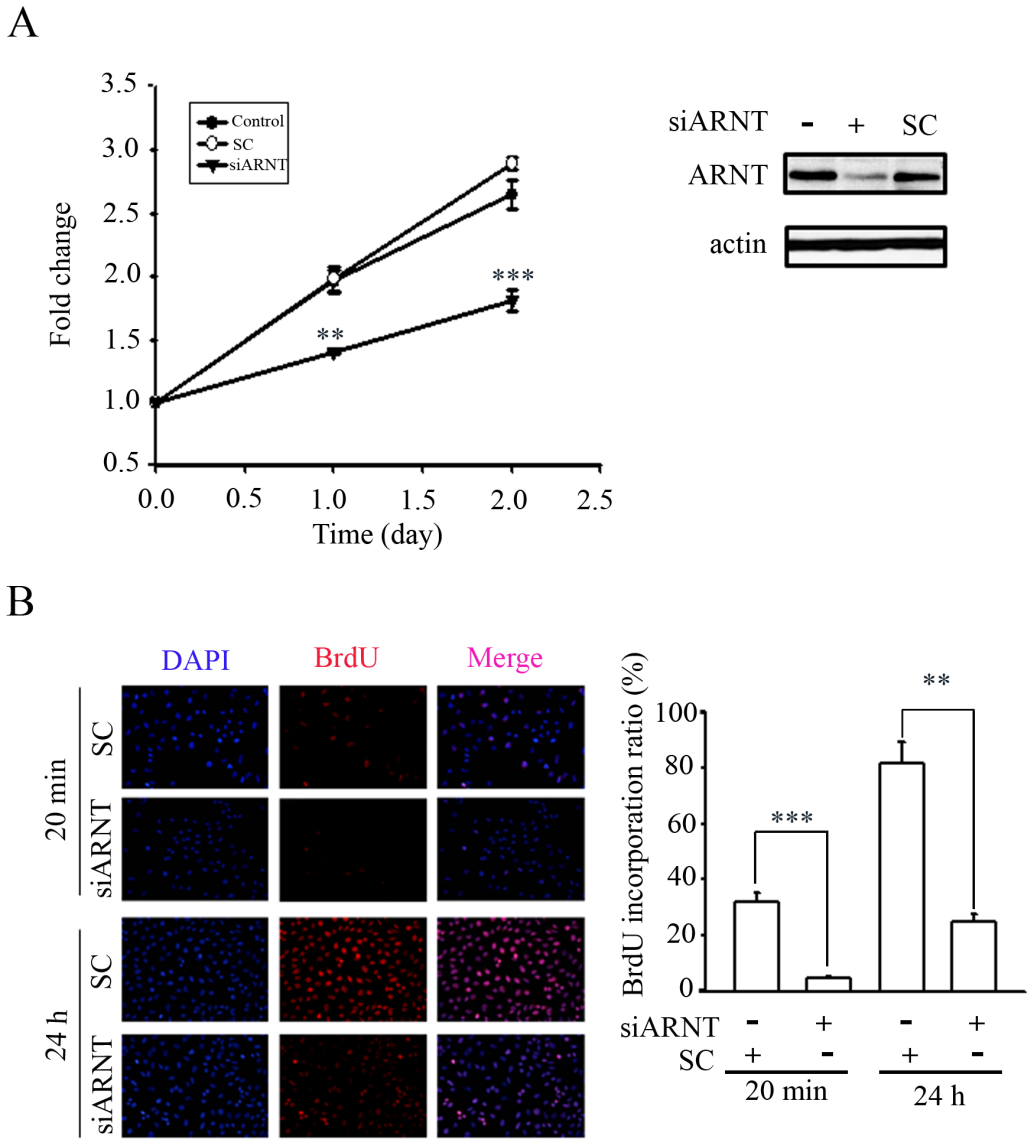
The ARNT regulates cell proliferation in a normoxic situation. (A) HeLa cells were transfected with 30 nM ARNT siRNA oligonucleotides and scramble oligonucleotides (SC) by lipofection. BrdU incorporation assay was performed as described in Materials and methods. Error bars denote mean + SD (n = 3). Statistical significance (***P<0.001; **P<0.01) between control and siARNT oligonucleatides-treated cells was analyzed by Student's *t* test. (B) HeLa cells were transfected with 30 nM ARNT siRNA oligonucleotides and scramble oligonucleotides (SC) by lipofection and labeled with 20 nM BrdU for 20 min or 24 h. Cells were fixed and stained with anti-BrdU antibodies followed by anti-mouse FITC and DAPI. The percentage of cancer cells with positive nuclear and BrdU staining was calculated by counting immunopositive cells in four randomly chosen. Error bars denote mean + SD (n = 4). Statistical significance (***P<0.001; **P<0.01) between control and siARNT oligonucleatides-treated cells was analyzed by Student's *t* test.

To clarify that ARNT is essential for tumor cell growth under normoxic conditions, cell proliferation was determined by a BrdU incorporation assay under an ARNT-knockdown condition. As shown in Fig. 1A, the cell proliferation rate was significantly reduced in siARNT cells. Results from pulse-labeling of BrdU (20 min) and synchronization of cells by thymidine block showed that siARNT reduced DNA synthesis (Fig. 1B) and retained cells in S-phase progression of the cell cycle (Fig. S1), respectively. These results suggested that ARNT regulates cell proliferation possibly through control of S-phase progression in normoxia.

### Knockdown of ARNT inhibits cell transformation

**Figure 2:**
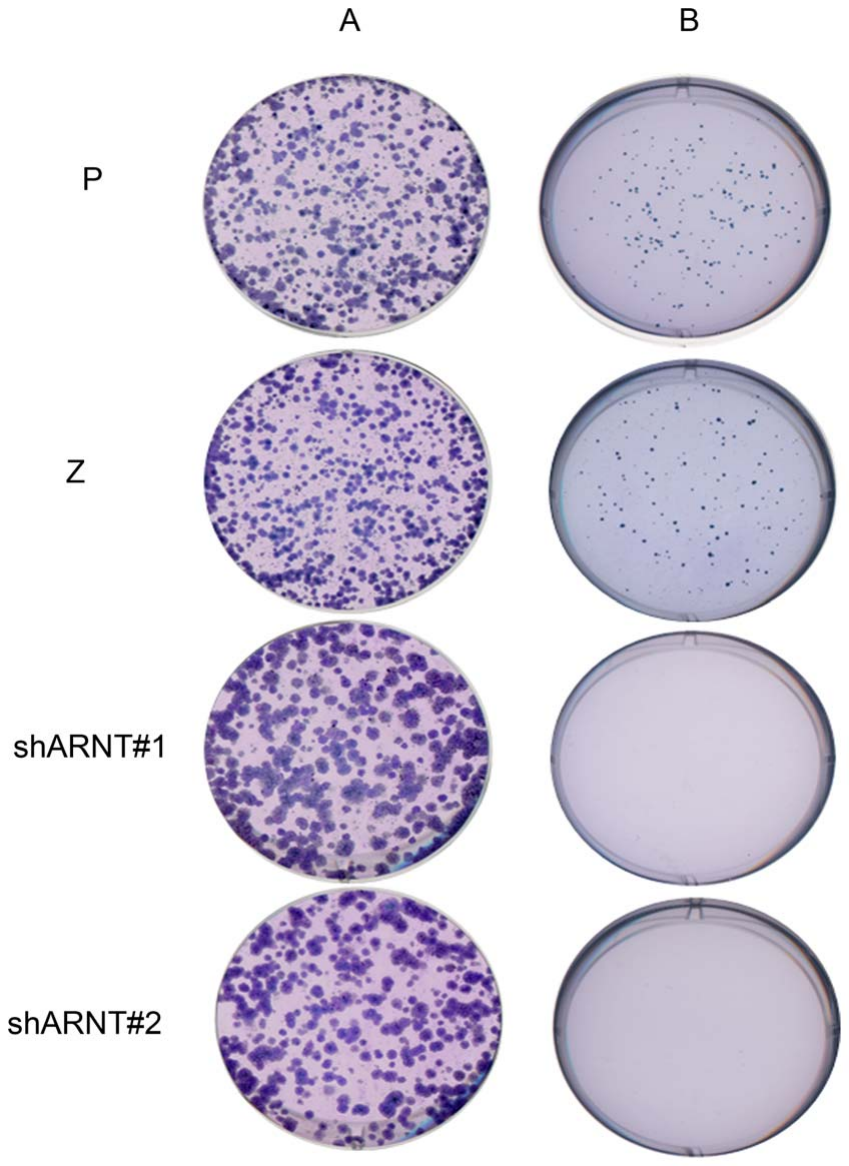
Knockdown of ARNT inhibits the proliferation and invasive ability of A375 cells. (A) Under a normoxic condition, A375 parental cells (P), shLacZ cells (Z), and ARNT-silenced cells (shARNT #1 and #2) were incubated in fresh culture medium in normoxic condition. After 7 days, cells were fixed and stained with Giemsa stain. The experiment was performed by colony formation assay. (B) The depletion of ARNT inhibits cellular transformation in cancer cells. Cells were seeded in the top layer of agar medium. After incubation for 3 weeks with supplemented medium, cells were fixed and stained with Giemsa. The experiment was performed by soft agar assay. Similar results were obtained in three independent experiments.

Based on the findings that ARNT expression enhances proliferation of cancer cells, the effect of ARNT on cell proliferation was further confirmed using a colony formation assay. As shown in Fig. 2A, although the size was not consistent with parental cells, numbers of colony decreased more obviously in ARNT-deficient cells. The reduced proliferation rate was also observed in ARNT deficient cells (Fig. S2). Interestingly, we found that the growth of shARNT cells was inhibited under 3D-culture condition (Fig. 2B), but not in 2D-culture conditions (Fig. 2A). These results indicated that depletion of ARNT reduced the ability of cellular transformation by cancer cells.

### ARNT-knockdown leads drug-resistant cells to be more sensitive to cisplatin

**Figure 3:**
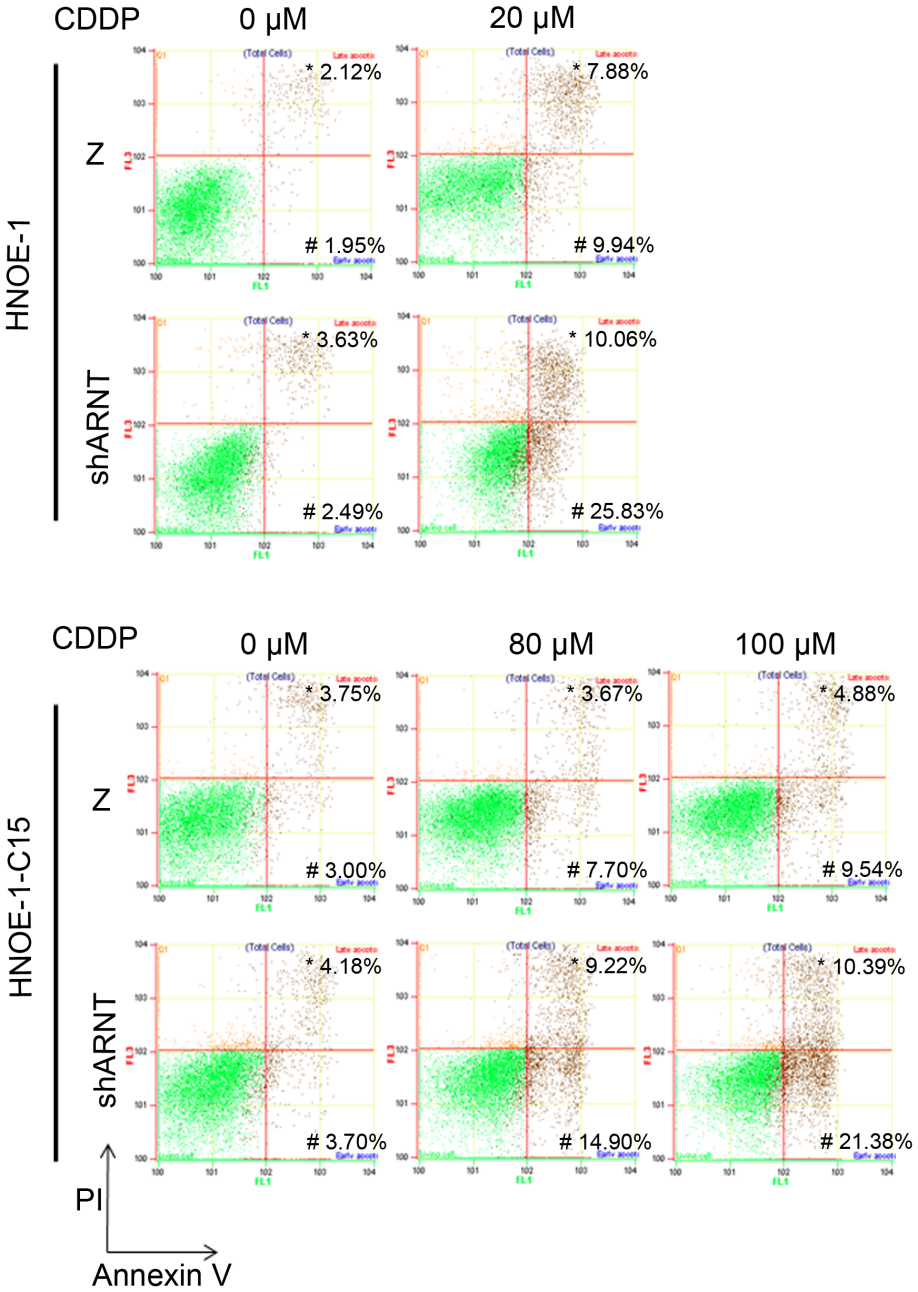
Knockdown of ARNT promotes cisplatin-induced cell apoptosis in both drug-sensitive and drug-resistant cells. HONE-1 shLacZ cells (Z), HONE-1 ARNT-deficient cells (shARNT), HONE-1-C15 shLacZ cells (Z), and HONE-1-C15 ARNT-deficient cells (shARNT) were treated with or without cisplatin (20 µM cisplatin for HONE-1 shLacZ cells and HONE-1 ARNT-deficient cells; 80 and 100 µM cisplatin for HONE-1-C15 shLacZ cells and HONE-1-C15 ARNT-deficient cells) for 24 h. Apoptotic cells were examined by staining with annexin V-FITC/propidium iodide (PI), and flow cytometry was used to analyze cell apoptosis. The apoptotic ratio was calculated. * late apoptosis; # early apoptosis. Similar results were obtained in three independent experiments.

**Figure 4:**
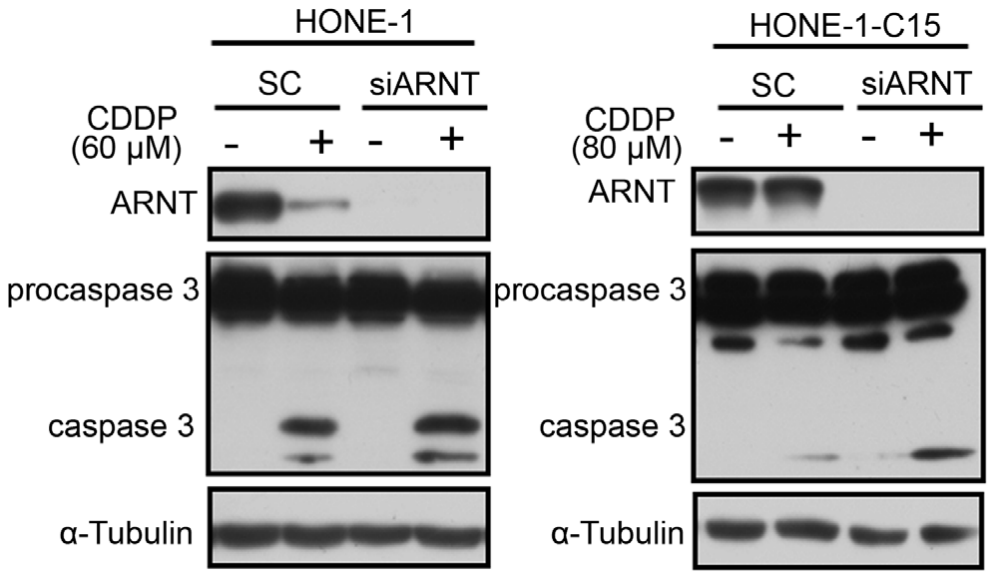
Knockdown of ARNT enhances cisplatin-induced activation of caspase-3 in drug-resistant cells. HONE-1 cells were transfected with 50 nM siARNT or 50 nM stealth RNAi negative control (SC) for 24 h and treated with or without cisplatin (60 µM for HONE-1 cells and 80 µM for HONE-1-C15 cells) in fresh cultured medium for 24 h. Total protein (including living cells and dead cells) was extracted, analyzed with Western blotting and detected with ARNT, caspase-3, and α-tubulin antibodies. Similar results were obtained in three independent experiments.

Among chemotherapeutic drugs, cisplatin induces cell death by forming cisplatin-DNA adducts which lead to DNA damage and impairs progression of the S-phase. The possibility that ARNT controls cell proliferation through regulating S-phase progression prompted us to examine whether it produced any effect on cisplatin-induced cell death. To clarify the importance of ARNT in regulating cisplatin resistance, we used a pair of cells, HONE-1 and HONE-1-C15 (HONE-1 cisplatin-resistant cells). Stable ARNT knockdown cell lines were generated by stably transfecting HONE-1 and HONE-1-C15 cell lines with the shARNT vector (Fig. S3). As shown in Fig. 3, cisplatin-induced apoptosis was more significant in shARNT cells than in parental HONE-1 cells. In addition, cisplatin caused less cell death in HONE-1-C15 cells than in HONE-1 cells, even with high-concentration treatment. However, HONE-1-C15 cells lost their resistance ability in an ARNT-knockdown condition (Fig. 3). Under cisplatin treatment, the ARNT was also degraded in parental HONE-1 cells, but not in resistant HONE-1-C15 cells (Fig. 4). Cisplatin promoted more caspase-3 activation in HONE-1 cells but not in HONE-1-C15 cells, even when a higher concentration of cisplatin was applied to HONE-1-C15 cells (Fig. 4). However, both HONE-1 and HONE-1-C15 cells became more sensitive to cisplatin when ARNT was deficient (Fig. 4). These results confirmed that expression of ARNT is a major factor regulating drug resistance by cancer cells.

### Chemotherapeutic drugs induce ARNT degradation and cell apoptosis

**Figure 5:**
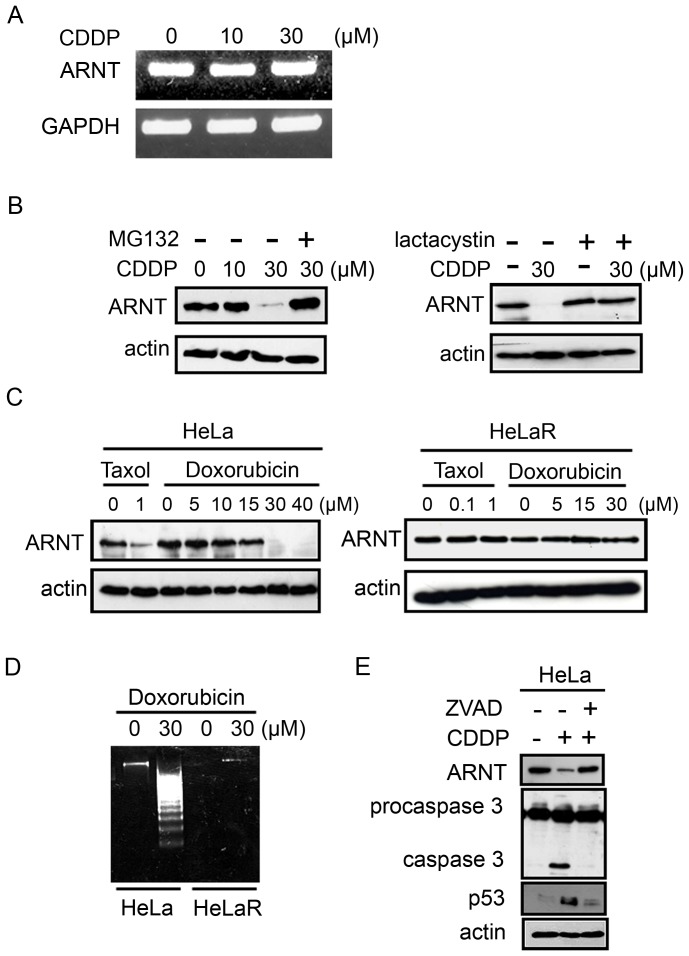
The effect of cisplatin on ARNT expression. (A) HeLa cells were treated with cisplatin for 24 h. Total RNA was extracted for reverse-transcription PCR with ARNT and GAPDH primers. (B) HeLa cells were treated with 10 µM MG132 or 1 µM lactacystin for 4 h followed by cisplatin for 36 h. Expression of ARNT and actin were analyzed by Western blotting analysis using anti-ARNT and anti-actin antibodies. (C) HeLa and HeLaR cells were treated with various concentrations of taxol and doxorubicin for 24 h. Expressions of ARNT and actin were detected by Western blotting analysis using anti-ARNT and anti-actin antibodies. (D) After 30 µM cisplatin treatment of HeLa and HeLa R cells, apoptotic DNA fragmentation was determined as described in "Materials and methods". (E) Cells were transfected with 30 nM ARNT siRNA oligonucleotides and scrambled oligonucleotides (SC) by lipofection. After 30 µM ZVAD treatment for 30 min followed by 30 µM cisplatin for 36 h, lysates of cells were prepared and subjected to SDS-PAGE and analyzed by Western blotting with antibodies against ARNT, procaspase-3, caspase-3, p53, and actin. Similar results were obtained in three independent experiments.

To clarify the mechanism involved in downregulation of ARNT in cisplatin-treated sensitive cells, expression of ARNT was further examined in various cancer cell lines. As shown in Fig. 5A, cisplatin produced no effect on the transcriptional level of ARNT messenger (m)RNA. However, cisplatin-induced ARNT deregulation was reversed in cells treated with a proteasome inhibitor (Fig. 5B). These results showed that cisplatin triggered ARNT degradation through proteasome-dependent pathways in sensitive cells. Interestingly, cisplatin-induced ARNT deregulation could also be reversed when different types of cancer cells were pretreated with the ROS scavenger NAC (Fig. S4). These results revealed that production of ROS, at least is one the causes to eliminate ARNT in cisplatin-treated cancer cells. To further examine whether deregulation of ARNT is also required for other chemotherapeutic drugs such as taxol and doxorubicin to induce cell death, HeLa and HeLa cisplatin-resistant (HeLa R) cells were treated with these drugs, and expressions of ARNT and fragmented DNA were studied. Doxorubicin and taxol inhibited ARNT expression and increased in fragmented DNA in HeLa cells (Fig. 5C & 5D). However, expression of ARNT was not changed in HeLa R cells after treatment with taxol or doxorubicin (Fig. 5C), and these results were consistent with the lack of fragmented DNA observed in HeLa R cells (Fig. 5D). In addition, shARNT cell lines were also more sensitive to cisplatin-induced cell death (Fig. 3). These results suggested that ARNT plays a pivotal role in contributing to resistance by cancer cells to various chemotherapeutic drugs. We also found that the caspase inhibitor, ZVAD, blocked the expression of p53 and activation of caspase-3 in cisplatin-treated cells (Fig. 5E). Interestingly, cisplatin-induced deregulation of ARNT was restored in ZVAD treated sensitive HeLa cells (Fig. 5E). In addition, cisplatin-induced caspase-3 activation, depletion of ARNT, and an increase of p53 were reversed in cells pre-treated with NAC (Fig. S4). These results indicated that cisplatin-induced activation of caspase-3 mediated the expression of ARNT and p53. ZVAD blocked caspase-3 and rescued ARNT expression in cisplatin-treated sensitive cells, suggesting that caspase-3 activation is essential for cisplatin-induced cell death in sensitive cells.

### The increase in ROS in ARNT-knockdown cells contributes to cisplatin-induced apoptosis

**Figure 6:**
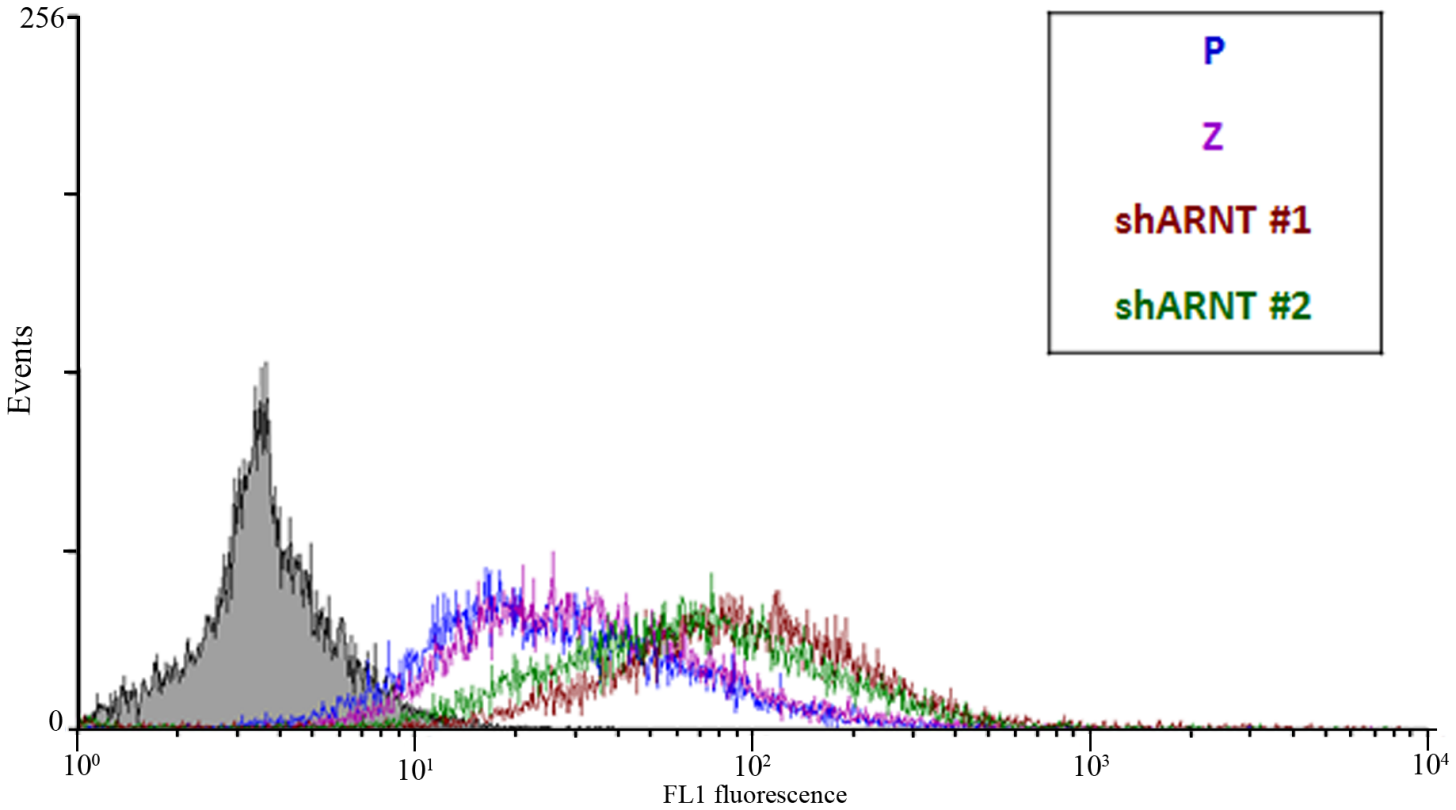
Knockdown of ARNT induces the amount of ROS. A375 parental and shARNT cells were incubated under a normoxic condition overnight and stained with carboxy-DCFDA to monitor ROS production. FL1 fluorescence indicated the fluorescence value of carboxy-2',7'-dichlorofluorescein diacetate (carboxy-DCFDA). Similar results were obtained in three independent experiments.

**Figure 7:**
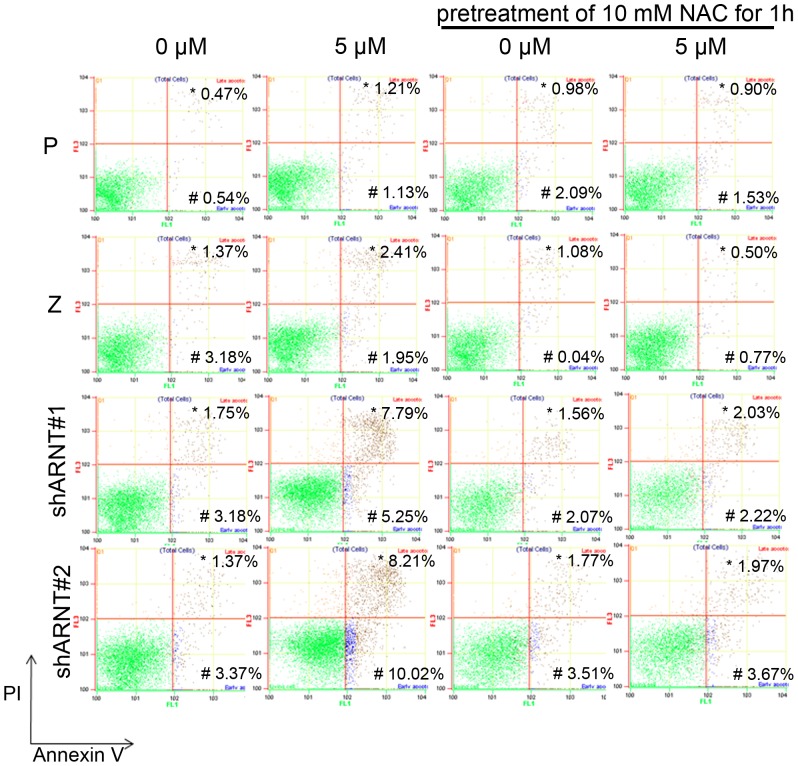
Depletion of ROS reduces cipaltin-induced shARNT cell apoptosis. Cells were pretreated with 10-acetylcysteine (NAC) for 1 h before A375 parental cells (P), shLacZ cells (Z), and ARNT-silenced cells (shARNT #1 and #2) were treated with 5 µM cisplatin, and then were incubated for 24 h. Apoptotic cells were examined by staining with annexin V-FITC/propidium iodide (PI), and flow cytometry was used to analyze cell apoptosis. The apoptotic ratio was calculated. * late apoptosis; # early apoptosis. Similar results were obtained in three independent experiments.

In addition to deregulating efflux pumps, an increase in the ROS level is another way for chemotherapeutic drugs to induce apoptosis. As shown in Fig. S4, depletion of ROS dramatically inhibited cisplatin-induced cell apoptosis. To further clarify the mechanism involved in ARNT-regulated cisplatin resistance, the role of ROS in cisplatin-induced cell death was examined. First, we identified the amount of ROS in parental (P), shLacZ (Z), and shARNT cells. Interestingly, we found that ROS was obviously higher in ARNT-deficient cells than in parental cells under normoxic conditions (Fig. 6 and Fig. S5A). The increase of ROS was reduced in shARNT cells treated with NAC (Fig. S5A). In addition, cisplatin-enhanced ROS amount was eliminated in cells treated with NAC (Fig. S5B). Cisplatin also induced higher production of ROS in shARNT cells (Fig. S5C), and the amount of ROS was reduced in cells treated with NAC. These results suggested that the expression of ARNT inhibited cisplatin-enhanced ROS production. To clarify whether the increase in ROS in shARNT cells plays a role in cisplatin-induced apoptosis, NAC was used to deplete ROS, and the apoptosis ratio was analyzed. As shown in Fig. 7, NAC significantly inhibited cisplatin-induced cell death in shARNT cells (Fig. 7). These results revealed that ARNT protected cancer cells from cisplatin-induced apoptosis, at least through reducing ROS production in cells.

## Discussion

Cancer therapeutic applications, such as radical treatment combined with chemo-therapeutic drugs mediated by induction of ROS, are major ways to eliminate cancer cells [22]. However, cancer cells are capable of resistance to the damage caused by ROS-induced apoptosis through alternative anti-apoptotic pathways, such as Akt, Kras, Braf, and Myc [23], [24]. In this study, we demonstrated that ARNT conferred an anti-apoptosis ability on cancer cells treated with the anti-cancer drug, cisplatin. Moreover, cisplatin produced greater ROS generation in ARNT-knockdown cells, resulting in enhancement of cell death. Similar to our findings that ARNT protected against cell damage by reducing ROS levels, leukemia cells were sensitive to troglitazone which also induces apoptosis through intracellular ROS [25]. Resistant leukemia cells exhibited an abundance of ARNT and also followed upregulation of SODs (SOD2), nuclear factor erythroid 2-related factor 2 (Nrf2) transcript, and intracellular glutathione concentration [25]. SOD2 reduces antioxidants and Nrf2 increases antioxidant enzyme activities [25]. In addition, Nrf2 regulates the transcription of mir125b to inhibit AhR repressor (AhRR), which protects the kidney from acute injury [26]. This indicates that formation of AhR/ARNT complexes may be regulated by mir125b [8]. These results suggest that ARNT may regulate SOD2 expression or ARNT/AhR complex formation to reduce cell injury caused by increased ROS.

As to regulation of ARNT, we found that cisplatin may inhibit the ARNT in some undiscovered ways. ARNT was degraded in a dose-dependent manner in non-resistant cells treated with cisplatin. ARNT's half-life seemed to be important for apoptosis caused by cisplatin. For example, proteasome inhibitors, such as MG132 and lactacystin, blocked the degradation of ARNT caused by cisplatin. However, cisplatin-resistant cancer cell lines showed that ARNT stability was not changed during treatment with cisplatin. These cancer cells with constant ARNT levels also showed a good ability to prevent ability of apoptosis. These results corresponded to a previous study in which ARNT disruption was correlated with proteasomal degradation via the ubiquitination process [27]. Consistent with our findings that ARNT was depleted by anti-cancer drugs, ARNT was also degraded by curcumin in normoxic and hypoxic conditions in various cancer cell types [27]. In addition, expression of ARNT can be restored by treatment with NAC, an ROS scavenger, in the presence of curcumin. These results are consistent with our findings that NAC rescued the expression of ARNT in cisplatin-treated sensitive cells. The ARNT can also be disrupted using H_2_O_2_ in cancer cells [27]. In addition to ROS and consistent with the fact that caspase-3 and caspase-9 also cleave ARNT at the Asp 151 amino acid site in vitro [11], we found that cisplatin-induced degradation of ARNT was repressed following treatment with the caspase inhibitor ZVAD. Thus, cisplatin-activated caspases may cause deregulation of ARNT in drug-sensitive cells but not in resistant cells. Chemotherapeutic drugs such as taxol and doxorubicin can also respectively induce cancer cell apoptosis through caspase-10 and p53 pathways [28], [29]. In this study, taxol and doxorubicin also enhanced ARNT degradation, resulting in apoptosis of sensitive cells. These results revealed that ARNT is an essential factor in protecting cancer cells against drug-induced damage. A previous study also revealed that cleavage of ARNT enhanced by hypoxia induced active caspase, and this downregulated transcriptional activity of survival genes induced by the HIF [27]. The production of ROS is one of effects through which cisplatin causes cell death [17]. It was also reported that mir-24 induced by ROS causes the depletion of ARNT in protein level in human hepatocellular carcinoma cell lines [30]. Taken together, ROS could be induced by depletion of ARNT, and then further produces negative feedback regulation of ARNT by induction of mirRNA. For the reason, the prevention of ARNT degradation in the initial treatment of drugs is important for survival of cancer cells. However, whether the ROS-induced mir-24 is the cause of suppression of ARNT in cisplatin-treated cells would be investigated in our further studies. Although, the mechanism involved in upregulation of ROS level induced by depletion of ARNT was not clear and would also be investigated, we speculated that the repression of ARNT could be one of mechanisms responsible for altering the level of ROS in cisplatin-induced cell death.

In general, ARNT can interact with HIF-1α to regulate genes involved in promoting angiogenesis, interact with c-Jun and Sp1 to modulate MDR1 expression, or regulate EGF-induced expression of COX-2, 12(S)-lipoxygenase, and p21*^WAF1/CIP1^*, which sense the change of microenvironmental component [1], [13]. ARNT also plays vital role in regulating tumor progression, detoxification, and efflux of anti-cancer drugs, which increases the survival chance in adverse circumstances [3], [8], [9], [24]. In chemotherapeutic treatment of cancer, cisplatin accumulates in cancer cells that causes DNA damage and induces apoptosis. The drug efflux pump, MDR1, is upregulated by ARNT to prevent cisplatin-induced apoptosis [9]. In addition to regulating drug resistance in previous study, ARNT also protects cells from microenvironmental toxicity. For example, the AhR/ARNT complex senses environmental toxins and regulates pathways responsible for detoxification. For example, TCDD induces numerous genes mediated by the AhR/ARNT complex, such as cytochrome P450 1A1 (CYP1A1) which can detoxify of polycyclic aromatic compounds [8], [31]. In this study, we found that ARNT plays further role in down-regulation of ROS to prevent cancer cells suffering damage from cisplatin treatment. Taken together, we speculated that the ARNT is a central mediator to eliminate cytotoxicity excited by environmental factors.

In conclusion, our results revealed that depletion of ARNT promoted in the effect of anti-cancer drugs on cancer cell death. In our understanding, there were at least two mechanisms involved in ARNT-caused drug resistance: MDR1 upregulation [9] and prevention of ROS production. Although the mechanism involved in regulation of ROS production by ARNT expression remains unknown, development of antagonists targeting the ARNT may provide new strategies for destroying resistant cancer cells.

## Materials and Methods

### Cell cultures

Cell lines of human malignant melanoma (A375) and human cervical cancer (HeLa) were purchased from American Type Culture Collection. Human nasopharyngeal carcinoma cells (HONE1 and HONE-1-C15) were kindly provided by Dr. Jane-Yang Chang (National Health Research Institutes, Taiwan) [32]. A375 and HeLa cell lines were maintained in Dulbecco's modified Eagle medium (DMEM) with 1% glucose (Gibco). HONE-1 cells and their derivative cisplatin-resistant variant, HONE-1-C15, were maintained in RPMI media 1640 (Gibco). All cell lines were supplemented with 10% fetal bovine serum (FBS, Hyclone), 100 µg/ml streptomycin (Sigma-Aldrich), and 100 U/ml penicillin (Sigma-Aldrich) in culture medium and incubated with 5% CO_2_ at 37 °C for maintenance. Cisplatin-resistant HONE-1-C15 cells were maintained in medium containing 15 µM cisplatin. The ARNT-deficient A375 cell lines were independent stable cell lines infected with lentiviral vector-derived ARNT small hairpin (sh)RNA [9]. The ARNT-deficient HONE-1 and HONE-1-C15 cell lines were stable cell lines infected with lentiviral vector-derived ARNT shRNA [9]. Drug-resistance schedules were used to develop the sublines resistant to cisplatin [9]. HeLa cells were exposed to cisplatin for a 9-month period. The resistance subline obtained by this procedure is denoted as HeLa R.

### Trypan blue exclusion assay

Cells were plated at 5×10^4^ per well in 24-well plates. After cells were allowed to attach overnight, the medium was replaced with fresh medium with or without cisplatin. Cells were incubated at 37 °C under a humidified hypoxic condition (1% O_2_) for an additional 24 h and trypsinized to resuspend them in medium containing serum. Trypan blue dye at 20 µl and 20 µl of a cell suspension were mixed to measure the numbers of living cells (with a dilution factor of 2). Stained cells were counted and regarded as dead cells.

### Bromodeoxyuridine (BrdU) incorporation assay

DNA synthesis in proliferating cells was detected by BrdU incorporation (Cell Signaling Technology, Danvers, MA). Parental and ARNT knockdown cells were spread onto 96-well plates and incubated for 24 h. 5-bromodeoxyuridine (BrdU) reagent was added to culture media for 0 to 48 h, 100 µl Fixing Solution was added to the cells for 30 min. The cells were washed with Wash Buffer and incubated for 1 h with 100 µl 1 x BrdU antibody. After adding 100 µl 1 x HRP-conjugate solution for 30 min, 100 µl TMB substrate solution was added. Following 30 min incubation, the stop solution was added. The OD was measured at 450 nm using a plate reader. For immunofluorescence, cells transfected with 30 nM ARNT siRNA oligonucleotides were pulse labeled with 20 nM BrdU for 20 min or 24 h. Cells were fixed and stained with anti-BrdU antibodies followed by anti-mouse FITC and DAPI. The percentage of cancer cells with positive nuclear and BrdU staining was calculated by counting immunopositive cells in four randomly chosen using Image J software.

### Colony formation assay

Cells were plated as separate single cells in 6-well cell-culture plates. After cells were allowed to attach overnight, cells were treated with or without 1 µM cisplatin for 8 h and the medium was changed to fresh culture medium. The culture medium was changed every 2 days. After incubation for 7 days, the cell colony was washed with PBS twice and fixed with 4% paraformaldehyde (PFA, Sigma-Aldrich). Methanol (JT Baker) was used to increase the penetration of cells, and Giemsa stain (Sigma-Aldrich) was used for cell staining. Colony numbers were determined with Image J software [33]. The colony formation was confirmed at least for three times.

### Soft agar assay

High-glucose DMEM containing 10% FBS with 0.5% agarose was plated on the bottom (1.5 ml/well) of 6-well plates. After the basal agar had congealed, 5×10^3^ cells were resuspended in high glucose DMEM containing 10% FBS with 0.25% agarose and layered on top (1.5 ml/well) of 6-well plates. Cells were incubated with 0.5 ml of supplemented medium after the top agar layer had congealed. The culture medium was changed every 2 days. After incubation for 2 weeks, cells were washed with PBS twice and fixed with 4% PFA. Methanol was used to increase the penetration of cells, and Giemsa stain was used for cell staining. The colony formation was confirmed at least for three times.

### Reverse transcription-PCR (RT-PCR)

Cell RNA was extracted by TriZol (Invriogen) reagent and followed by manuscript. Two µg RNA was reversed by ImProm-II™ reverse transcriptase (Promega) and used for cDNA template of polymerase chain reaction (PCR). 50 ng cDNA template was used for PCR with 2.5U Tag DNA polymerase (MD Bio, Taiwan) [34]. The primer sets were used as following: ARNT (F): 5′-TGGGTCCAGCCATTGCCTCT-3′, ARNT (R): 5′-CGAGCCAGGGCACTACAGGT-3′, GAPDH (F): 5′-CCATCACCATCTTCCAGGAG-3′, GAPDH (R): 5′-CCTGCTTCACCACCTTCTTG-3′. The PCR reactions were then electrophoresis by 2% SeaKem LE agarose gel (Lonza, ME, USA).

### Western blot analysis

Cells were harvested with 1x CCLR or RIPA buffer (10 mM Tris buffer at pH 6.5, 150 mM NaCl, 50 mM EDTA, 1% DOC, and 1% NP-40 containing protease inhibitors). The protein concentration of cell lysates was determined with a BCA protein assay kit (23225, Thermo) and iMark microplate absorbance reader (Bio-Rad). Proteins were resolved on a 10% sodium dodecylsulfate (SDS)-polyacrylamide gel for electrophoresis and transferred to polyvinylidene fluoride (PVDF) membranes. Non-fat dry milk (5%) in PBS containing 0.1% Tween-20 (PBST) was used for blocking. After washing three times with PBST, the PVDF membranes were incubated with primary antibodies overnight at 4°C. After primary antibodies were removed, PVDF membranes were washed four times with PBST and incubated with horseradish peroxidase (HRP)-conjugated secondary antibodies for 1 h at room temperature. Membranes were washed with PBST three times and incubated with an enhanced chemiluminescent HRP substrate (Millipore) for x-ray film detection. The antibodies used in this study were as follows: ARNT (sc-17811, Santa Cruz), α-tubulin (Sigma-Aldrich), and caspase-3 (9665S, Cell Signaling). The expression of protein was confirmed at least for three times.

### RNA interference (RNAi) transfection

Cells were transfected with Duplexed Stealth RNAi against ARNT [9] and stealth RNAi negative control (SC, Invitrogen) using serum-free Opti-MEM medium and lipofectamine RNAiMAX transfection reagent. Small interfering (si)RNA was incubated with lipofectamine RNAiMAX (siRNA: lipofectamine RNAiMAX of 25 nM: 1 µl) and serum-free Opti-MEM medium for 15 min at room temperature before being transfected into cells. After 8 h, medium was changed to serum-free high glucose DMEM.

### DNA fragmentation assay

Cells that were or were not treated with doxorubicin were collected and washed with PBS; lysed in a solution containing 10 mM Tris HCl at pH 8.0, 10 mM EDTA, and 0.5% Triton X-100; digested with 0.1 mg/ml RNase A at 37 °C for 1 h; and then centrifuged at 12,000x*g* for 25 min to pelletize the chromosomal DNA. The supernatant was digested with 1 mg/ml proteinase K at 50 °C for 2 h in the present of 1% SDS, extracted with phenol and chloroform, precipitated in cold ethanol, and subjected to electrophoresis on 1.5% agarose gels containing 0.5 µg/ml ethidium bromide. DNA fragments were visualized by ultraviolet light transillumination. Photographs were taken with the aid of a computer-assisted image processor.

### Flow cytometry to detect apoptosis

Cells from different conditions were trypsinized and combined with cells in the medium by centrifugation. Harvested cells were washed with PBS and incubated with annexin V binding buffer containing annexin V-FITC/propidium iodide (PI, 556547, BD) at room temperature for double staining for 15 min. Flow cytometry was used to analyze cell apoptosis with a Cell Lab Quanta SC Flow Cytometer (Beckman Coulter). The experiment was repeated for three times.

### Flow cytometry to analyze ROS

Cells were incubated overnight at 37 °C under humidified normoxic conditions or hypoxic condition (1% O_2_). After being washed with PBS, cells were incubated with 0.1 µM 5-(and-6)-carboxy-2',7'-dichlorofluorescein diacetate (carboxy-DCFDA, C369, Invitrogen) in serum free medium for 30 min at 37°C for staining and then replaced serum-containing culture medium for cell recovery for 15 min. Flow cytometry was used to analyze ROS production with a Cell Lab Quanta SC Flow Cytometer by using wavelength of 525 nm band pass. The experiment was repeated for three times.

### Statistical analysis

In all experiments, statistical significance was analyzed by Student's *t* test. P<0.05 was considered significant.

## Supporting Information

Figure S1 **S phase progression delayed in ARNT knockdown cells.** HeLa cells were transfected with 30 nM of ARNT siRNA oligonucleotides and scrambled oligonucleotides (negative control) by lipofectamine, and then were synchronized at G_1_/S phase by treating 2 mM thymidine for 19 h. After refreshed culture medium, cells were collected at certain time point after release and cell cycle was analyzed by flow cytometry. (A) Results of one of three independent experiments were shown. S phase progression was shown in parental and ARNT knockdown cells. (B) Percentage of each phase in cell cycle progression in parental and ARNT knockdown cells was calculated. (C) The expression of ARNT and actin was analyzed by Western blotting with antibodies against ARNT and actin.
(TIF)

Figure S2 **The proliferation rate was reduced in ARNT deficient cells.** A375 cells were transfected with 30 nM of ARNT siRNA oligonucleotides and scrambled oligonucleotides by lipofectamine. The cell numbers were counted by trypan blue exclusion assay. Statistical significance (*P<0.05; **P<0.01) between control and siARNT oligonucleatides-treated cells was analyzed by Student's *t* test. Data shown are the means ± SD of three independent experiments.
(TIF)

Figure S3 **Knockdown of ARNT in HONE-1 and HONE-1-C15 cells.** Proteins of ARNT and α-tubulin were analyzed by Western blotting and respectively detected with ARNT and α-tubulin antibodies. Similar results were obtained in three independent experiments.
(TIF)

Figure S4 **NAC prevents the degradation of ARNT induced by cisplatin.** A375, HONE1 and HeLa cells were pretreated with 20 mM NAC, and then treated with 30∼60 µM cisplatin for 24 h. ARNT, capase3, p53 and actin protein level were detected by Western blotting. Three independent experiments were performed.
(TIF)

Figure S5 **NAC depletes the amount of ROS in cisplatin-treated cells.** (A) A375 and HONE1 parental and ARNT knockdown (shARNT) cells were treated with 20 mM NAC for 25 h. Flow cytometry was used to analyze ROS production as described in Material and methods. (B) HeLa, A375 and HONE1 cells were treated with 20 mM NAC for 25 h, and then treated with 30 µM cisplatin for 24 h. Flow cytometry was used to analyze ROS production as described in Material and methods. (C and D) A375 and HONE1 parental and ARNT knockdown (shARNT) cells were treated with 20 mM NAC for 25 h, and then treated with 30 µM cisplatin for 24 h. Flow cytometry was used to analyze ROS production as described in Material and methods. The image was depicted by FlowJo software. Similar results were obtained in three independent experiments.
(TIF)
