# Supplementary figures and images for: An Increase in Reactive Oxygen Species by Deregulation of ARNT Enhances Chemotherapeutic Drug-Induced Cancer Cell Death
Source: PLoS One. 2014 Jun 12;9(6):e99242. doi: 10.1371/journal.pone.0099242 (PMC4055634; doi:10.1371/journal.pone.0099242)

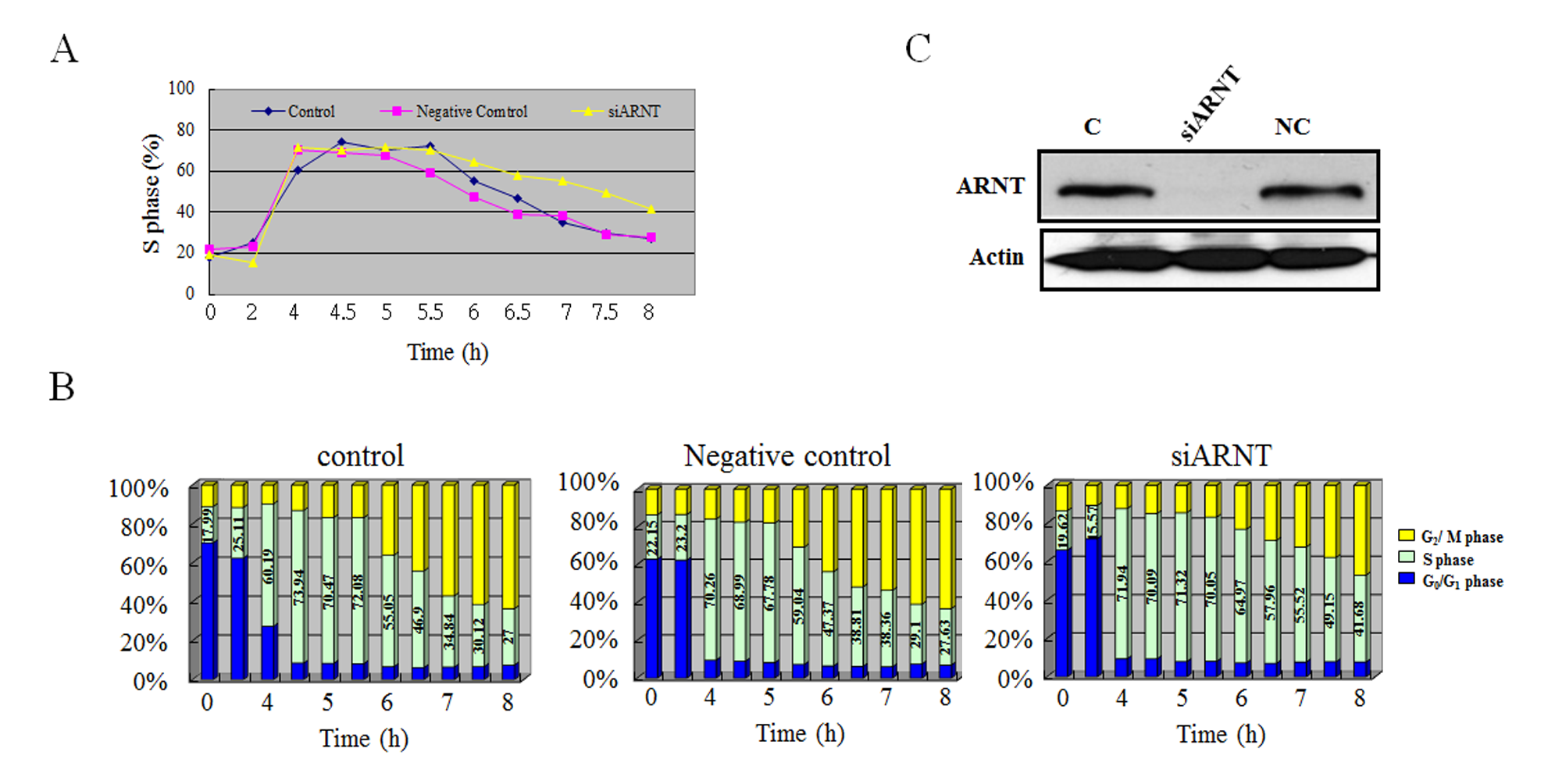

Supplement: Figure S1 — S phase progression delayed in ARNT knockdown cells. HeLa cells were transfected with 30 nM of ARNT siRNA oligonucleotides and scrambled oligonucleotides (negative control) by lipofectamine, and then were synchronized at G1/S phase by treating 2 mM thymidine for 19 h. After refreshed culture medium, cells were collected at certain time point after release and cell cycle was analyzed by flow cytometry. (A) Results of one of three independent experiments were shown. S phase progression was shown in parental and ARNT knockdown cells. (B) Percentage of each phase in cell cycle progression in parental and ARNT knockdown cells was calculated. (C) The expression of ARNT and actin was analyzed by Western blotting with antibodies against ARNT and actin. (TIF) [file pone.0099242.s001.tif]

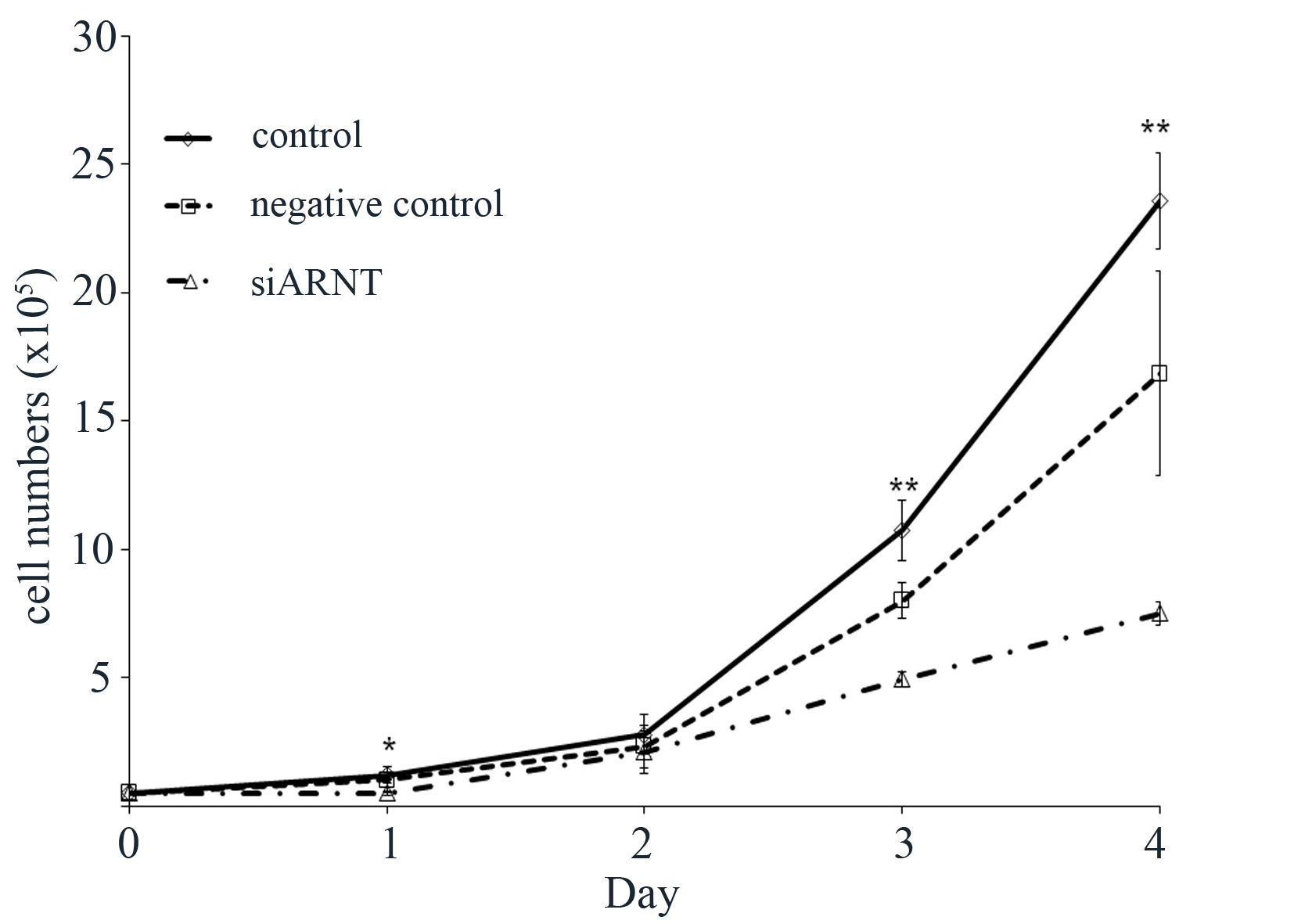

Supplement: Figure S2 — The proliferation rate was reduced in ARNT deficient cells. A375 cells were transfected with 30 nM of ARNT siRNA oligonucleotides and scrambled oligonucleotides by lipofectamine. The cell numbers were counted by trypan blue exclusion assay. Statistical significance (*P<0.05; **P<0.01) between control and siARNT oligonucleatides-treated cells was analyzed by Student's t test. Data shown are the means ± SD of three independent experiments. (TIF) [file pone.0099242.s002.tif]

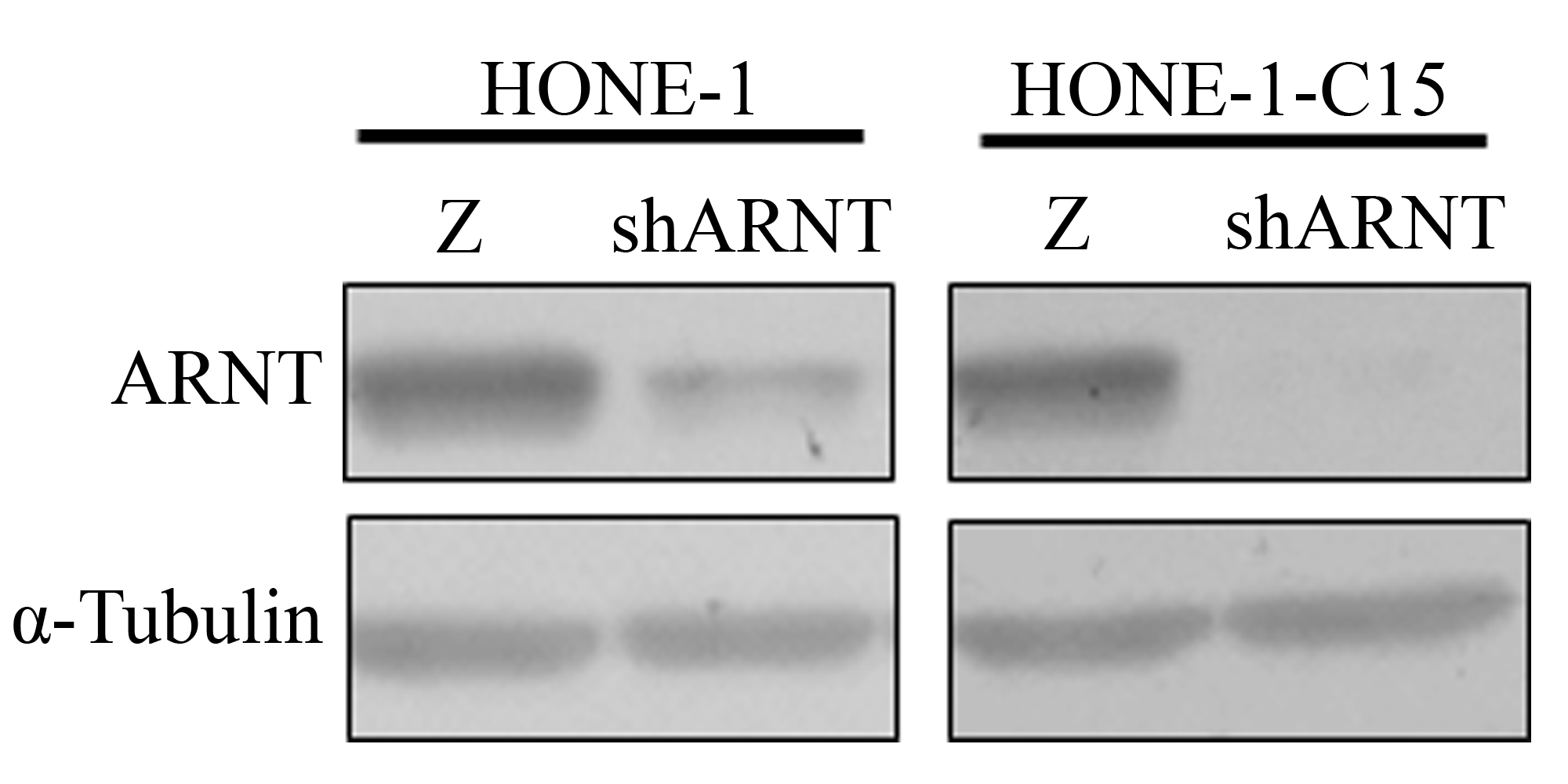

Supplement: Figure S3 — Knockdown of ARNT in HONE-1 and HONE-1-C15 cells. Proteins of ARNT and α-tubulin were analyzed by Western blotting and respectively detected with ARNT and α-tubulin antibodies. Similar results were obtained in three independent experiments. (TIF) [file pone.0099242.s003.tif]

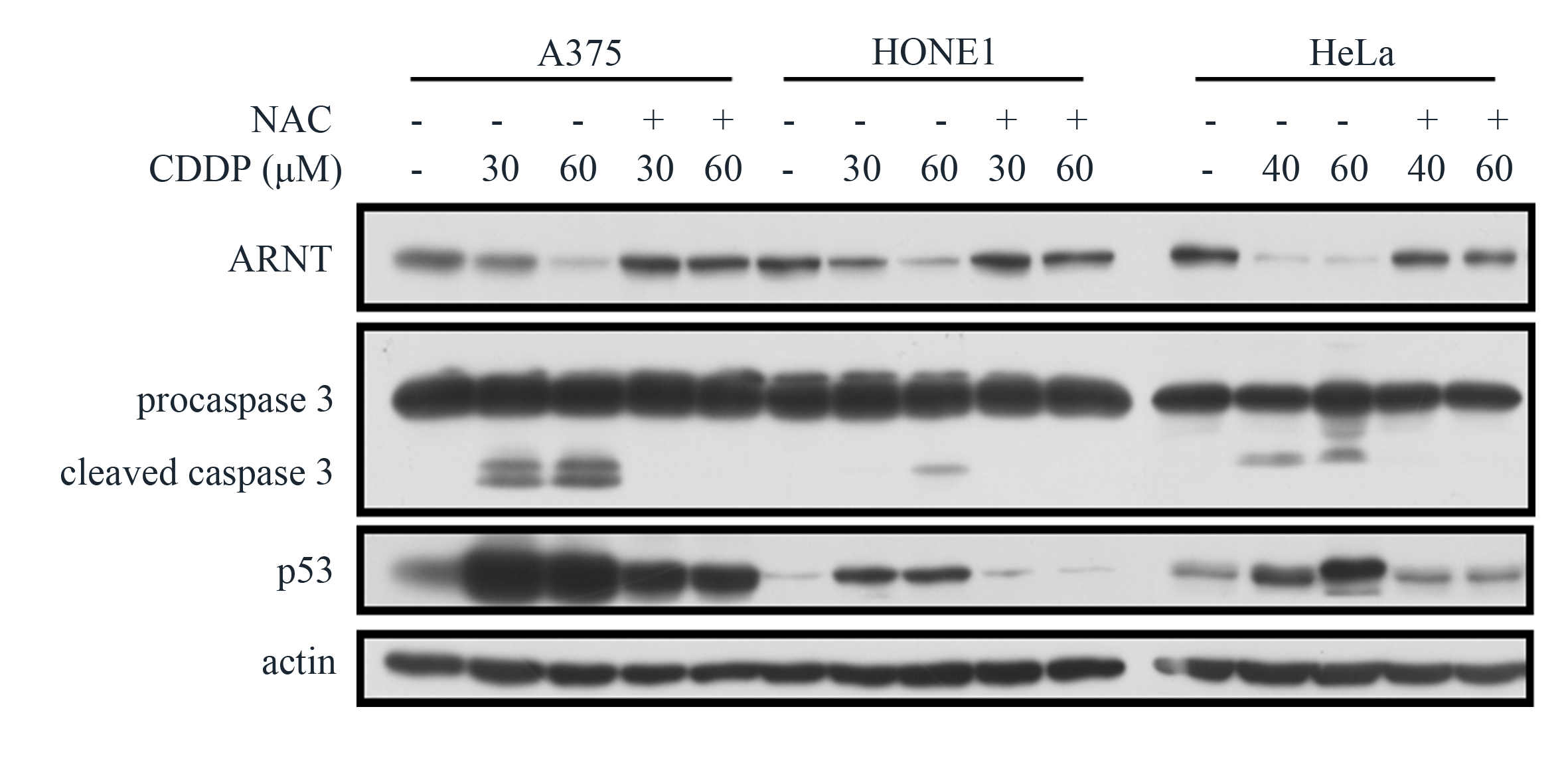

Supplement: Figure S4 — NAC prevents the degradation of ARNT induced by cisplatin. A375, HONE1 and HeLa cells were pretreated with 20 mM NAC, and then treated with 30∼60 µM cisplatin for 24 h. ARNT, capase3, p53 and actin protein level were detected by Western blotting. Three independent experiments were performed. (TIF) [file pone.0099242.s004.tif]

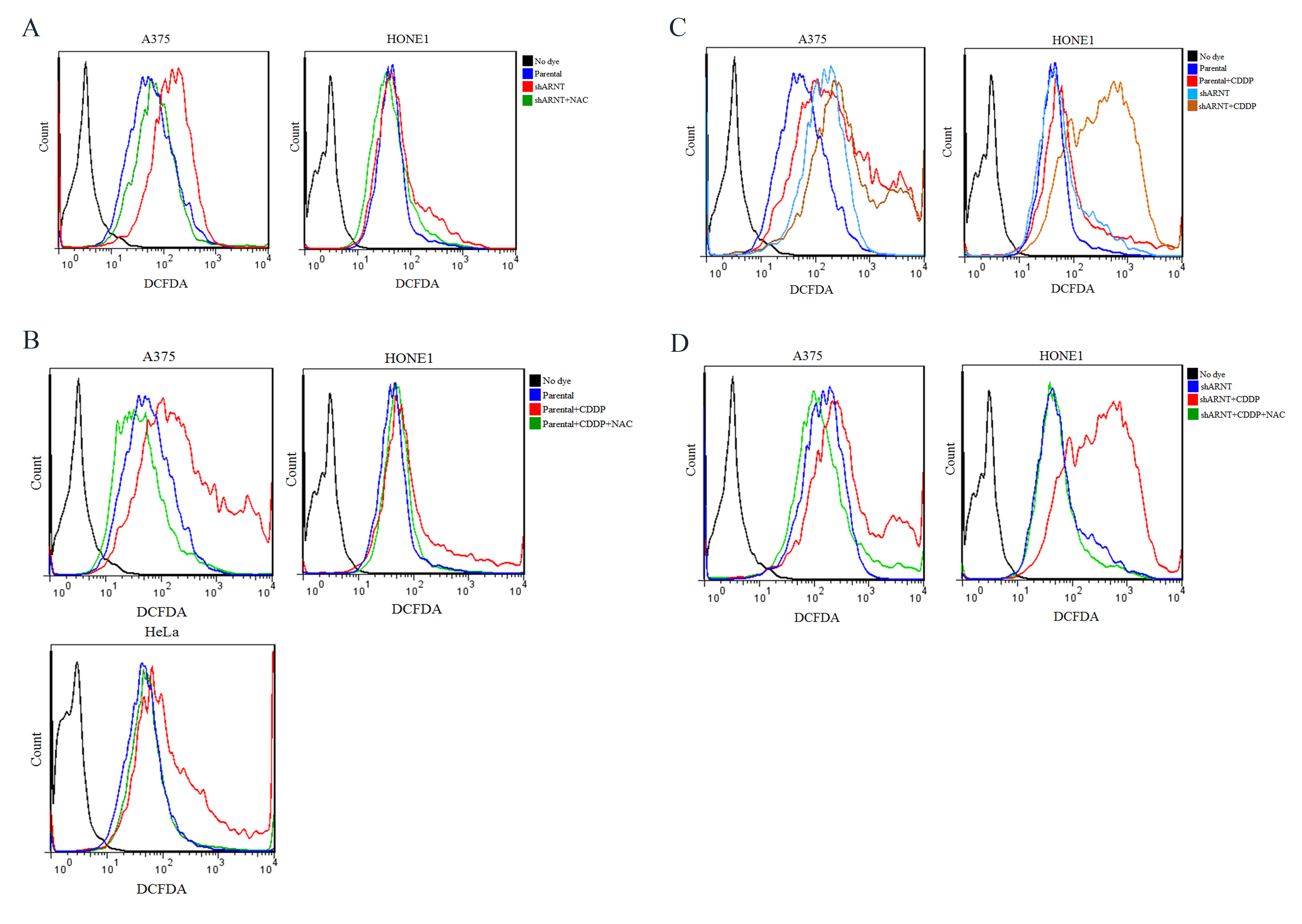

Supplement: Figure S5 — NAC depletes the amount of ROS in cisplatin-treated cells. (A) A375 and HONE1 parental and ARNT knockdown (shARNT) cells were treated with 20 mM NAC for 25 h. Flow cytometry was used to analyze ROS production as described in Material and methods. (B) HeLa, A375 and HONE1 cells were treated with 20 mM NAC for 25 h, and then treated with 30 µM cisplatin for 24 h. Flow cytometry was used to analyze ROS production as described in Material and methods. (C and D) A375 and HONE1 parental and ARNT knockdown (shARNT) cells were treated with 20 mM NAC for 25 h, and then treated with 30 µM cisplatin for 24 h. Flow cytometry was used to analyze ROS production as described in Material and methods. The image was depicted by FlowJo software. Similar results were obtained in three independent experiments. (TIF) [file pone.0099242.s005.tif]
